# Physical and Sensory Properties of Mayonnaise Enriched with Encapsulated Olive Leaf Phenolic Extracts

**DOI:** 10.3390/foods9080997

**Published:** 2020-07-24

**Authors:** Federica Flamminii, Carla Daniela Di Mattia, Giampiero Sacchetti, Lilia Neri, Dino Mastrocola, Paola Pittia

**Affiliations:** Faculty of Bioscience and Technology for Agriculture, Food and Environment, University of Teramo, Via Balzarini 1, 64100 Teramo, Italy; fflamminii@unite.it (F.F.); gsacchetti@unite.it (G.S.); lneri@unite.it (L.N.); dmastrocola@unite.it (D.M.); ppittia@unite.it (P.P.)

**Keywords:** olive leaf polyphenols, encapsulation, functional food, mayonnaise, alginate/pectin beads, phenolic extract, food enrichment

## Abstract

This work aimed to study the physical, structural, and sensory properties of a traditional full-fat mayonnaise (≈ 80% oil) enriched with an olive leaf phenolic extract, added as either free extract or encapsulated in alginate/pectin microparticles. Physical characterization of the mayonnaise samples was investigated by particle size, viscosity, lubricant properties, and color; a sensory profile was also developed by a quantitative descriptive analysis. The addition of the extract improved the dispersion degree of samples, especially when the olive leaf extract-loaded alginate/pectin microparticles were used. The encapsulated extract affected, in turn, the viscosity and lubricant properties. In particular, both of the enriched samples showed a lower spreadability and a higher salty and bitter perception, leading to a reduced overall acceptability. The results of this study could contribute to understanding the effects of the enrichment of emulsified food systems with olive by-product phenolic extracts, both as free and encapsulated forms, in order to enhance real applications of research outcomes for the design and development of healthy and functional formulated foods.

## 1. Introduction

Nowadays, the increasing amount of attention being paid to health and wellbeing has modified consumer choices toward foods that, besides meeting nutritional requirements and providing hedonistic gratification, may also guarantee benefits by exerting desired functional properties. Mayonnaise is a traditional sauce prepared by the gentle mixing of oil with egg yolk, vinegar, salt, and spices to form an oil-in-water emulsion with a dispersed lipid phase ranging between 60 and 80%. It represents one of the most consumed sauces worldwide and is highly appreciated for its special flavor and creamy mouthfeel.

The words “functional” and “mayonnaise” do not seem to get along; however, if such a combination of words is considered from another point of view, it could lead to an innovative and challenging functional food [1]. Several strategies have been proposed to make mayonnaise a healthier product: along with the reduction of fat, which is the most widespread approach used up to now, a more recent approach is based on the enrichment of emulsified sauces with beneficial ingredients that can respond to the health-related needs of people (antioxidant, prebiotics, and probiotics). Moreover, from a food science point of view, functional ingredients, such as natural antioxidants, are known to improve the oxidative stability of the product and allow the replacement of synthetic and debated antioxidants. The interest in the use of natural ingredients is often associated with the opportunity of recovering functional and bioactive compounds from food waste and by-products, such as those derived from the olive oil chain. Recently, several studies have proposed the enrichment of different food products with an olive leaf phenolic extract (OLE), as in the case of vegetable oils, due to their antioxidant activity (i.e., sunflower, soybean, maize, and frying oils) [2], or meat products against oxidative [3] and microbial spoilage [4]. Moreover, the use of OLE was also tested in cereal [5] and dairy products [6]. Apart from having healthy, antioxidant, and antimicrobial properties, olive polyphenols have been proven to exert important technological functionality, such as a water/oil holding capacity and emulsifying activity [7], and can represent a useful multifunctional ingredient that can help in the production and stabilization of complex food products such as emulsions. However, consumers’ willingness to accept foods produced with olive by-product ingredients depends on the perception of different factors, mainly related to the general attitude of the consumer, rather than a product-specific choice. Indeed, information about the characteristics of olive by-products and the perception of the benefits from sustainable consumption can possibly offset the consumers’ choice with a positive association between the use of vegetable by-products and sustainable production and environmental responsibility [8].

One main barrier to a large usage in food manufacturing is that phenolic-rich extracts are characterized by chemical and physical instability under conditions commonly encountered in processing and storage (temperature, oxygen, and light) [9], as well as by an unpleasant bitter and pungent taste [10].

Therefore, encapsulation could represent a valid strategy for overcoming all of these drawbacks and various investigations have been carried out in this field by using different technologies and approaches [11,12,13,14]. Nonetheless, up to now, few works have reported the use of encapsulated OLE in foodstuffs: In one study, an OLE encapsulated by nano-emulsions was added in soybean oil, increasing the solubility and controlling the release of olive leaf phenolic compounds, as well as enhancing its antioxidant activity when compared to non-encapsulated OLE and synthetic antioxidants [15]. In another study, OLE encapsulated in water-in-oil-in-water double emulsions (W_1_/O/W_2_) was used in meat systems, improving the oxidative stability of the product, while providing a healthier lipid profile with respect to the free extract [16]. Furthermore, the enrichment of cereal, dairy, beverages, and spreadable products with OLE encapsulated with different techniques was assessed, in order to improve their shelf-lives [17,18,19,20], but additional studies are needed, in order to widen the knowledge on the qualitative properties of OLE-enriched formulated products.

Therefore, the aim of this research was to study the physical, structural, and sensory properties of a full-fat mayonnaise (≈ 80% oil) enriched with an olive leaf phenolic extract (OLE), encapsulated in alginate/pectin microparticles. The physical characterization of the mayonnaise samples after preparation was investigated by measuring the particle size, viscosity, tribology, and color; a sensory profile was also developed by a quantitative descriptive analysis. For comparison purposes, samples enriched with pure OLE and without OLE (control) were prepared. The results will provide a new formulation and technological insights on the effect of the enrichment of a full-fat mayonnaise with encapsulated OLE on its physical and structural properties and, at the same time, evaluate the effect of encapsulation on OLE performances.

## 2. Materials and Methods

### 2.1. Materials

A dried olive leaf extract (OLE) with a standardized concentration of oleuropein (40%) was kindly donated by Oleafit srl (Isola del Gran Sasso, Teramo, Italy) Dried OLE-loaded alginate/pectin microcapsules (Alg/Pec) with an average volume weighted mean diameter (D_4,3_) of 62.6 ± 0.2 μm were obtained by applying an emulsion/internal gelation technique [11]. Sunflower oil, eggs, salt, vinegar, and lemon juice were purchased in a local supermarket. All of the reagents used were of analytical grade.

### 2.2. Mayonnaise Preparation

Mayonnaise samples were prepared according to a reference recipe with the following formulation: Oil (500 g ≈ 78%), five egg yolks (27 g ≈ 12%), vinegar (10 g ≈ 2%), salt (5 g ≈ 1%), lemon juice (37.5 g ≈ 7%), and sodium azide 0.05% (*w*/*w*). The samples were prepared using a lab-scale mixer (Bimby TM31, Vorwerk, Wuppertal, Germany) in a two-step standardized process: Eggs, vinegar, salt, and lemon juice were preliminary mixed (100 g·min, 1 min), and oil was then slowly added under vigorous mixing (from 500 up to 1000 g·min) for 20 min, allowing complete oil incorporation. In order to evaluate the exploitability of pure OLE and Alg/Pec + OLE beads as functional ingredients, a final concentration of 200 mg of total phenolic compounds per kg of product was added to mayonnaise. In particular, ≈ 1 g/kg (≈ 0.09%) of OLE and ≈ 4 g/kg (≈ 0.32%) of Alg/Pec + OLE, both in a dried form, were added to the water phase during the preliminary mixing (first step). Three following series of samples were thus prepared: A control (without phenolic extract added), Mayo + OLE (enriched with pure OLE), and Mayo + Alg/Pec (enriched with Alg-Pec + OLE microspheres). Just after preparation, the following evaluations were carried out on the samples: Particle size distribution, microstructure, color, viscosity, tribology, and sensory profile evaluations.

### 2.3. Particle Size

Particle size distribution was measured by laser diffraction analysis and the use of a particle size analyzer (Mastersizer Hydro 3000, Malvern Instruments Ltd., Worcestershire, UK). Mayonnaise samples (0.2 g) were diluted with 20 mL of a 1% (*w*/*v*) sodium dodecyl sulphate (SDS) solution, gently stirred with a magnetic stirrer until complete dispersion [21], and thereafter added to the Hydro 2000S dispersion unit containing distilled water at 2500 rpm until an obscuration of 5–6% was reached. Each sample was analysed in triplicate and five records for each measurement were collected. The optical properties of the sample were defined as follows: Refractive index of 1.46 and absorption of 0.00. Droplet size measurements are reported as particle size distribution curves (PSD), the surface mean diameter (D_3,2_), and the volume mean diameter (D_4,3_).

### 2.4. Microstructure

An Olympus BX53F optical microscope (Olympus, Tokyo, Japan) was used to evaluate the mayonnaise’s microstructure. An amount of each sample was deposited until a very thin and homogeneous layer developed on the slide and images were obtained with 100× magnification and acquired with a QCAM fast 1934 (QImaging, Surrey, BC, Canada), equipped with a 55 mm objective. Images were elaborated through the Software “Image Pro Plus 7.0” (Media cybernetic, Inc., Rockville, MD, USA).

### 2.5. Color Evaluation

The color of the mayonnaise was evaluated with a CHROMA METER CR5 instrument (Konica Minolta, Osaka, Japan), illuminant D65; each sample was homogeneously distributed into a glass vessel and the color was recorded at five different points. The average of the five measurements was assessed for all the colorimetric parameters (L*, a*, and b*). In the CIE Lab colour space, L* represents the lightness within the range of 0 (black) to 100 (white); a* is the redness, from green (−a*) to red (+a*); and b* is the yellowness, from blue (−b*) to yellow (+b*). The a* and b* parameters were used to calculate the tonality angle (Hue angle, *h°*) according to Equation (1):

(1) $$h^{{}^{\circ}}=tan^{-1}\left(\frac{b^{*}}{a^{*}}\right)$$

### 2.6. Flow Behavior and Tribological Measurements

Mayonnaise rheological measurements were performed at 25 °C by a controlled stress–strain rheometer (MCR 302, Anton Paar, Graz, Austria) connected to a circulating water bath for temperature control. The flow behavior of the samples was evaluated by using a parallel plate configuration (50 mm) and a gap distance of 1 mm, and excess sample protruding from the edge of the sensor was carefully trimmed off with a thin blade. Flow curves were measured by recording viscosity values shearing the samples at logarithmic increasing shear rates from 3 to 300 s^−1^. Flow curves were built for all of the tested samples and fitted to the Herschel–Bulkley model by using Excel’s solver software to obtain the yield stress (τ_0_), consistency index (k), and flow index (*n*). The friction and lubricant properties of model-like mayonnaise samples were evaluated using a tribology apparatus (Anton Paar, Graz, Austria) fitted to the rheometer. The measuring cell consisted of a ball-on-three-plates geometry consisting of a rotating glass ball and three stationary cylindrical polydimethylsiloxane (PDMS) pins fixed to the sample holder. The ball measured 12.7 mm in diameter and the pins measured 6 mm in diameter and height. The friction and lubricating properties of mayonnaise samples in between two surfaces were recorded at 30 °C with a normal force (FN) of 1 N. The Stribeck curves for each sample were shaped by plotting the friction factor (−) versus the sliding velocity (Vs).

### 2.7. Sensory Evaluation

Sensory evaluation was carried out through quantitative descriptive analysis (QDA) by a panel consisting of 15 assessors. The panelists were students and technical staff of the Faculty of Bioscience and Technology for Agriculture, Food and Environment (University of Teramo, Teramo, Italy). Before analysis, brief training was carried out, in order to verify and discuss the vocabulary and to explain the scales being used. The following attributes were evaluated: Spreadability, consistency, and color uniformity for the appearance and structure; saltiness, bitterness, sourness, and astringency for flavor and taste; creaminess and grittiness for texture and mouthfeel; and then the overall acceptability. The ranking was defined as follows: 1 = lowest intensity and 10 = highest intensity. The samples (about 10 g) were served at room temperature in white plastic dishes with teaspoons. Judges were asked to first observe all of the samples, in order to evaluate the appearance and structure, and then, to put the mayonnaise into their mouth and evaluate the other attributes (flavor, taste, texture, and overall acceptability). Water was used for cleaning the mouth between tasting different samples. Data were normalized to the average score given by each panelist, for each attribute. Then, data were recalculated using the calibration curve obtained by plotting each attribute’s raw data (*y*-axis) versus normalized data (*x*-axis). Through this approach, differences in the scale of values of each non-calibrated panel member were avoided. The average scores for each attribute were determined and reported as spider plots.

### 2.8. Statistical Analysis

All of the data are the average of at least three measurements and reported as the mean and corresponding standard deviation; for the viscosity parameters, the median was considered. One-way ANOVA analysis was applied to experimental data. Linear regression analyses were carried out on data and the goodness of fit of the models was checked by the determination coefficient R^2^. Tukey’s test was used to establish the significance of differences among the mean values at the 0.05 significance level. Data were processed with XLSTAT software (Addinsoft SARL, New York, NY, USA).

## 3. Results and Discussion

The effect of the enrichment with Alg/Pec + OLE microparticles was studied by evaluating several physical properties of the mayonnaise samples just after preparation. In particular, the particle size, viscosity, tribology, and color were determined for their effect on the structural properties and stability of the emulsified structure and the sensory attributes.

### 3.1. Particle Size and Microstructure

In Table 1, the particle size of the mayonnaise samples, expressed as both D_4,3_ and D_3,2_, which are related to the size of the bulk of the droplets constituting the mayonnaise and the size of small droplets [22], respectively, are reported together with the specific surface area (SSA).

**Figure 1:**
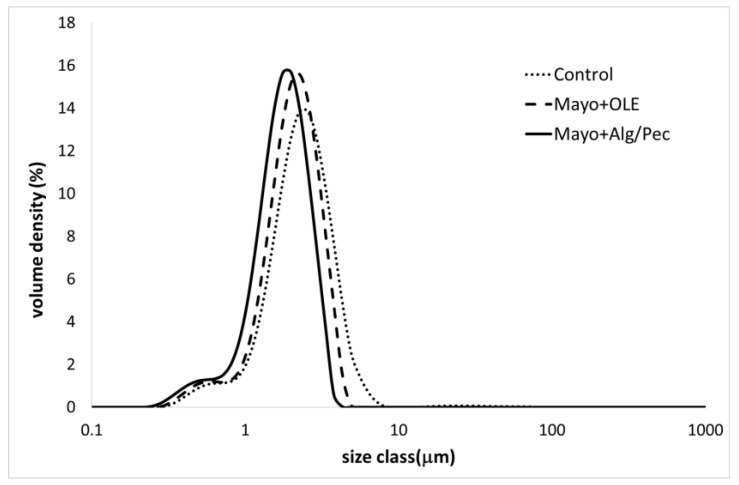
Particle size distribution curves of the differently enriched mayonnaise samples after preparation.

The results showed a reduction of the volume mean diameter (D_4,3_) and the surface mean diameter (D_3,2_) values for both of the enriched samples (Mayo + Alg/Pec and Mayo + OLE), with respect to the control, and a corresponding increase of the specific surface area with values of 4272, 3663, and 3270 for Mayo + Alg/Pec, Mayo + OLE, and the control, respectively. In general, the particle size distribution curves of the samples showed a polymodal distribution with two main populations and a shift towards a lower size in both of the enriched systems, which was more evident in the Mayo + Alg/Pec sample (Figure 1).

The decrease of the particle size in both of the enriched systems can be associated with the presence of olive leaf amphiphilic phenolic compounds, which have been proven to lower the oil/water interfacial tension and exert emulsifying properties, especially when tested in acidic conditions (acetate buffer, pH 4.5) [7]. In the case of Mayo + Alg/Pec, the presence of microparticles could have further improved the formation and stabilization of the emulsified structure, likely due to a Pickering stabilizing mechanism. Indeed, particles are able to stabilize oil/water interfaces, making them mechanically stronger, and are able to provide a sufficient steric repulsion force to inhibit droplet coalescence during emulsification [23]. The positive effect of OLE on the dispersion degree of the oil droplets is in accordance with other authors who prepared mayonnaise samples enriched with increasing amounts of olive phenolic extract [24]. In particular, the mayonnaise prepared with sunflower oil (SO) with an amount of oil (≈ 80%) comparable to the systems under investigation in this study, showed quite similar values of D_4,3_ with respect to the control samples. However, when the enrichment was carried out at a similar concentration (200 ppm) to the one used in this work, a less broad particle distribution with a lower degree of polydispersity was obtained. Moreover, in the same study, olive oil mayonnaise-like emulsions formulated with different naturally phenolic-rich extra virgin olive oils displayed similar results when a low phenolic olive oil (270 mg/kg) was used as the source of phenolic compounds. Therefore, such results strengthen the argument for the use, for the formulation of high oil content emulsions, of a phenolic-rich extract recovered by olive by-products, with respect to a more expensive ingredient, such as extra virgin olive oil.

**Figure 2:**
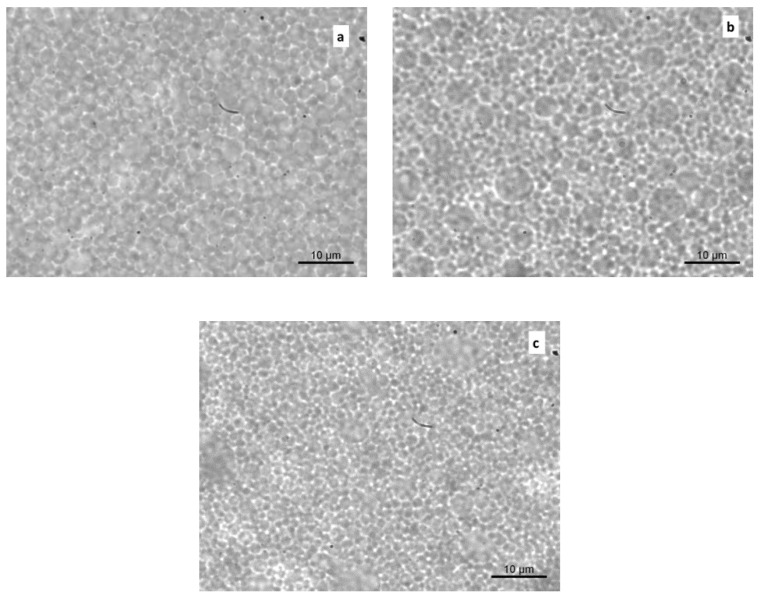
Optical micrograph images of mayonnaise samples just after preparation. Control (**a**), Mayo + OLE (**b**), and Mayo + Alg/Pec (**c**).

Optical microphotographs were captured to characterize the microstructural features of the samples (Figure 2a–c). In general, a well-dispersed oil phase could be observed in all of the systems, in agreement with the particle size results. While the presence of larger droplets was evident in samples a and b, defining a higher degree of polydispersity, a more finely dispersed structure and a closely packed distribution of oil droplets were visible in the mayonnaise enriched with OLE-loaded alginate/pectin microparticles.

### 3.2. Color Properties

**Figure 3:**
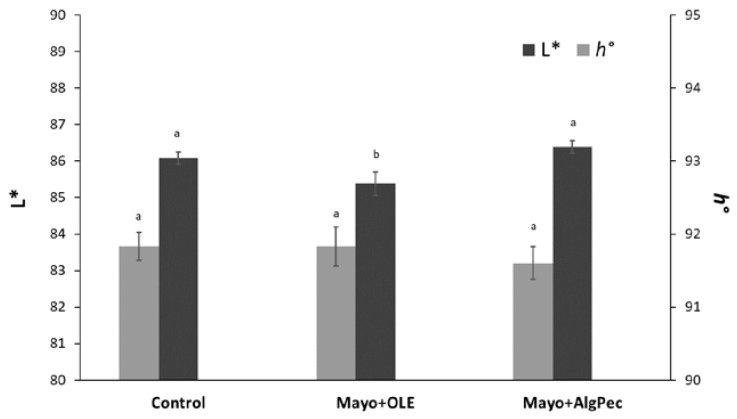
Color properties in terms of the lightness (L*) and hue angle (*h°*) of the mayonnaise samples under investigation. Different letters for parameters are significantly different by Tukey’s HSD test (*p* < 0.05).

Color has a main impact on consumers’ choice, as it is one of the most important sensory features that affect the willingness to purchase or taste a food. In general, the typical pale yellow color of mayonnaise originates from the egg yolk and oil and may be further influenced by the addition of mustard, additives, or some other spices with coloring effects (i.e., Annatto E160 and Turmeric). The enrichment of mayonnaise with unconventional ingredients, which are different with respect to those used in a standard recipe, could lead to physical and chemical changes that may affect, in turn, the color of the final products. In Figure 3, the chromatic indices of lightness and hue angle (L* and *h°*, respectively) of mayonnaise samples, prepared with the addition of free OLE or OLE encapsulated in alginate/pectin microparticles, are reported, along with the control sample.

The lightness value (L*), which is the colorimetric parameter referring to the ability of the system to reflect and scatter the light, was, in general, similar to data reported in the literature for full-fat mayonnaise [25,26,27]. Significant differences (*p* < 0.05) between the L* value of the control and Mayo + Alg/Pec sample with respect to that of the Mayo + OLE sample were observed and this result may reflect the different dispersion degrees of the systems. In particular, the Mayo + OLE product displayed a significantly lower lightness due to the color of the added OLE extract, which, in the case of Mayo + Alg/Pec samples, was mitigated by the encapsulation.

On the contrary, the results of the hue angle were close to 90°, confirming the yellow color, without significant differences (*p* > 0.05) among the samples. Overall, these results highlight that the addition of the olive leaf extract, either as a pure or encapsulated form, did not alter the visual appearance of the emulsion.

### 3.3. Effect of OLE Enrichment on Flow Behavior and Lubricant Properties

The viscosity properties of concentrated emulsions, such as mayonnaise, are strictly related to the close packing of the dispersed oil droplets as they interact with one another in the matrix: The closer the droplets, the higher the viscosity, as a consequence of the higher droplet–droplet interaction [24].

**Figure 4:**
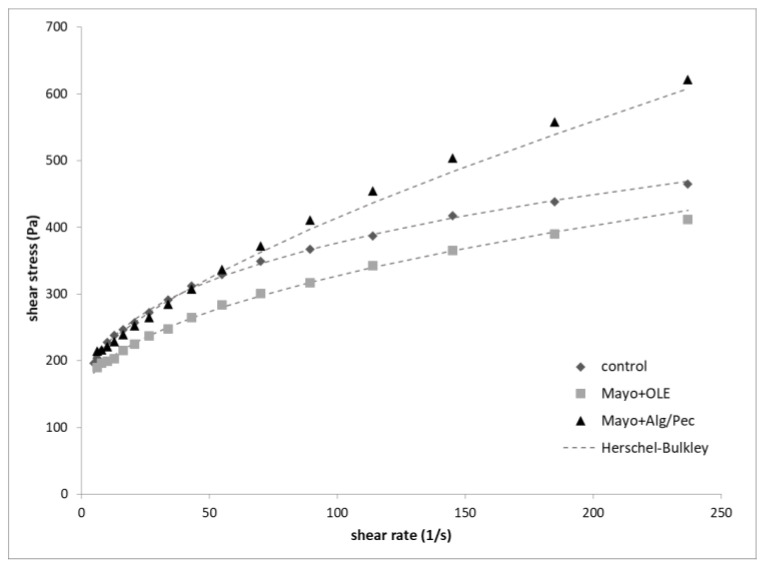
Flow curves of the mayonnaise after preparation.

The flow curves of mayonnaise samples are reported in Figure 4. In general, all of the mayonnaise samples exhibited a non-Newtonian shear-thinning behavior and a yield point related to the initial resistance of the systems to flow. Control and Mayo + OLE samples are both characterized by a similar trend, with a tendency to break-down at a higher shear rate (300 s^−1^). At a similar shear rate, OLE-enriched mayonnaise displayed lower shear stress values with respect to the control, while the Alg/Pec mayonnaise samples showed higher yield stress and shear stress values. This latter behavior may be related to several factors, including the higher packing degree of the lower size oil droplets (Figure 2c) and the thickening effect of beads swelling due to the presence of alginate and pectin in the aqueous continuous phase. Recent studies observed an increase in viscosity for mayonnaise enriched with carbohydrate-based capsules due to the thickening effect of the polymers (i.e., dextran, pullulan, glucose syrup, and zein) used as shell material upon their disintegration [28,29]. Moreover, it must be pointed out that the pH values of the samples were 3.87 ± 0.06, 3.92 ± 0.01, and 3.89 ± 0.01 for the control, Mayo + OLE, and Mayo + Alg/Pec, respectively, which are values close to the average isoelectric point of egg yolk proteins, when the protein charge is minimized and both the viscoelasticity and stability of mayonnaise are generally enhanced [30].

Mayonnaise flow curves were fitted with the Herschel–Bulkley model, which is commonly used to describe the flow properties of concentrated emulsified systems such as mayonnaise [31]; the flow parameters yield stress *τ*_0_ (Pa), consistency index K (Pa s^n^), and flow index *n* of the samples after preparation are listed in Table 2.

When compared to the control, both of the enriched samples were statistically different (*p* < 0.05) in terms of K, *τ*_0_, and *n*, with Mayo + Alg/Pec showing the highest *τ*_0_ and *n* values. As reported in the literature, yield stress values are an index of the strength of the attractive forces between the oil droplets [24,32] and this is in agreement with the results related to the Mayo + Alg/Pec sample, which exhibited the finest oil dispersion (Table 1).

Moreover, all of the mayonnaises presented a flow index (*n*)—the parameter that describes a pseudoplastic shear thinning behavior—lower than 1. It is interesting to note that the flow index tended to increase from the control to the enriched samples, with Mayo + Alg/Pec mayonnaise showing the highest value, corresponding to a lower pseudoplastic behavior. A shear-thinning profile in an oil-in-water emulsion is an indication that the system flows more readily as the dispersed particles align with the direction of flow; the enrichment with alginate/pectin OLE-loaded beads, which undergo swelling upon hydration, caused an increase of solids in the dispersing phase that likely hindered the alignment of the oil droplets.

**Figure 5:**
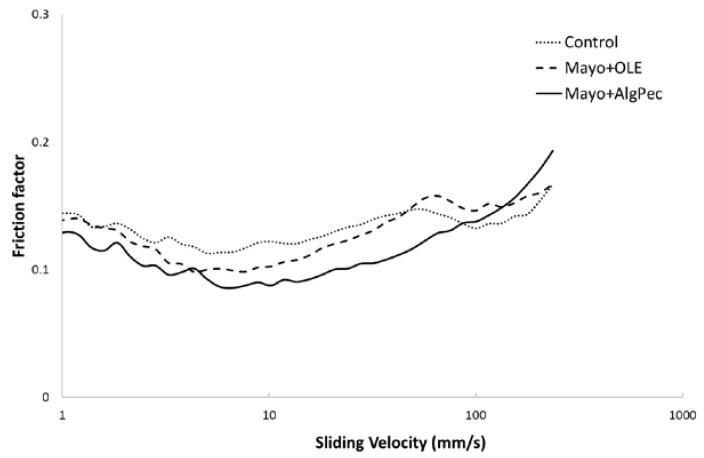
Stribeck curves of mayonnaise samples.

It is known that flow behavior can affect the mouthfeel of food emulsions, while other sensory attributes, such as creaminess, can be correlated with the lubrication properties of the emulsified structure. Overall, the perception of a food emulsion in the mouth is initially dominated by the bulk viscosity at the front of the oral cavity, where the gap between the tongue and palate is rather large (>10 μm) [33]; upon chewing, the product mixed with the saliva is squeezed between moving surfaces, such as the tongue and palate (or food substrate and palate), and the oral perception depends on its thin film rheological behavior. To better analyse the rheological properties of the mayonnaise samples in the form of thin films, tribological investigations were carried out. The lubrication properties of the control and differently enriched mayonnaise samples were represented in the form of a Stribeck curve (Figure 5), where the friction coefficient (−) was plotted against the sliding velocity (mm s^−1^) in the regions from 1 mm s^−1^, generally associated with mouth-like conditions during food consumption, up to 200 mm s^−1^, which is the maximum speed that the human tongue can move [34].

For all samples, it is possible to observe the occurrence of a typical Stribeck curve where the different regimes can be distinguished: In particular, the boundary regime, at low sliding speeds, with surface–surface interactions dominating the friction response with high friction factors; the mixed regime, at mid-speeds, when the sample starts to separate from the contact surfaces, decreasing the friction; and, finally, the hydrodynamic regime, where the surfaces are completely separated by the fluid with an increase of the friction values. For all of the mayonnaise samples, a similar behavior against the entertainment speed was noticed, with some differences in the friction factor values with the following ranking: Mayo + Alg/Pec < Mayo + OLE < control, and a clear discrimination among samples. With the exception of the highest sliding speeds, where all of the samples present similar trends, the mayonnaise enriched with Alg/Pec microparticles showed the lowest friction values, probably associated with a higher content of solids in the formulation and the finest oil droplet dispersion, with a corresponding better lubricant behavior. The highest lubricant properties of Mayo + Alg/Pec could also be attributed to the swelling of the hydrogel particles, which made them more deformable, allowing a decrease in the friction [35]. Another interesting result is related to the fact that Mayo + Alg/Pec sample exhibited a shift of the transition from a mixed to hydrodynamic regime toward a higher speed with respect to control and Mayo + OLE samples.

Despite the general effect of the addition of OLE, in both the free form and encapsulated in Alg/Pec beads, on the lubricant properties, which decreased the friction factors obtained by tribological measurements, additional investigations are needed to describe the behavior of such systems during oral processing, where other parameters, including the presence of saliva and mouth conditions (e.g., temperature), may play a key role.

### 3.4. Effect of OLE Enrichment on the Sensory Profile

**Figure 6:**
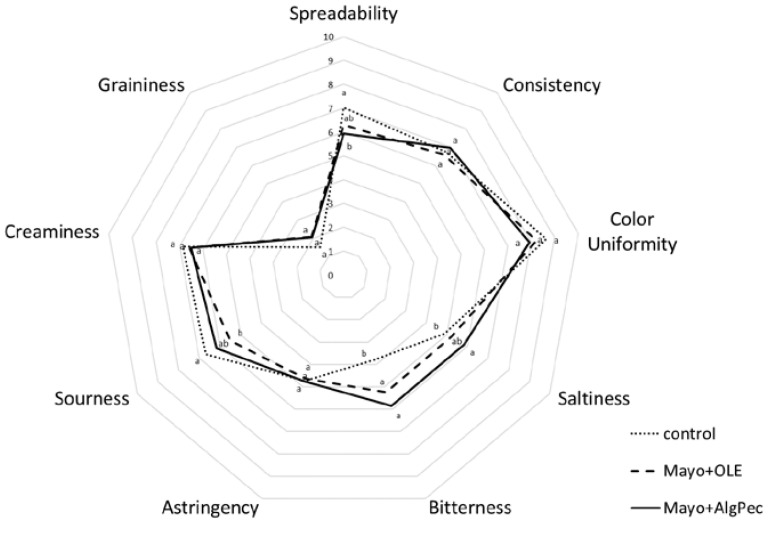
Spider plot of specific sensory attributes of the mayonnaise formulations. Lowercase letters for each attribute indicate significant differences (*p* < 0.05).

Mayonnaises enriched with pure OLE or OLE encapsulated in alginate/pectin microparticles were assessed by a group of 15 panelists using a quantitative descriptive analysis. Before each session, the assessors were trained on each attribute, the scale used, and how to score the samples. The average scores of each selected attribute are reported in the spider plot shown in Figure 6. As expected, polyphenols addition, either in a pure or encapsulated form, significantly affected the bitterness perception with respect to the control sample, while no marked differences were reported for the two enriched mayonnaise samples, which were both perceived with a medium bitterness intensity (score ≈ 5.8) not appreciated by the assessors. The enrichment of mayonnaise did not influence the color, astringency, consistency, creaminess, and graininess parameters, which could be related to the presence of OLE or microcapsule powder. The saltiness, sourness, and spreadability were partially influenced by the addition of alginate/pectin microcapsules. It can be hypothesized that the saltiness increase may have been caused by the presence of calcium alginate of the microgels structure; moreover, in the literature, it is reported that bitter compounds such as caffein can enhance the effect of salt [36]. However, to the best of the authors’ knowledge, to date, no literature data has been reported on the interaction between the saltiness and bitterness of olive leaf phenolic compounds. The enriched samples scored low in spreadability with respect to the control, indicating some resistance to spread, which is a result that is in accordance with the higher values of yield stress observed in the flow curves. Eventually, the sensory evaluation highlighted that both of the enriched mayonnaise samples, but in particular the system fortified with OLE-loaded microparticles, displayed the lowest overall acceptability from the panel. The results reflect the low ability of alginate/pectin microparticles to mask the bitterness of an OLE extract and present new challenges for further investigations, especially in terms of alternative encapsulation methods, in order to overcome such issues.

## 4. Conclusions

The enrichment of mayonnaise with OLE-loaded microparticles (Mayo + Alg/Pec) was effective in improving its physical properties in terms of both the dispersion degree of the oil droplets and lubricant properties of the emulsions; however, the highest viscosity, which in turn was reflected by the lowest spreadability, led to a different sensory perception with respect to both the control and Mayo + OLE samples. The addition of OLE, either as pure extract or encapsulated in alginate/pectin microparticles, slightly affected the chromatic parameters with respect to the control sample. The sensory evaluation depicted a lower overall acceptability for both of the enriched samples, likely due to the increased salty and bitter perception. The encapsulation technique adopted was thus not able to mask or control the bitterness perception, leading to the conclusion that further investigations are needed in terms of encapsulation methods, bead compositions and structures, and mayonnaise formulations to overcome such issues. The proposed research could represent a valid contribution in the wide panorama of functional foods and opens new opportunities for formulating healthier foodstuffs while valorizing olive by-products.

## Figures and Tables

**Table 1:** Particle size parameters of mayonnaise samples after preparation, as affected by olive leaf phenolic extract (OLE) and alginate/pectin (Alg/Pec) + OLE enrichment.

	D [4;3] μm	D [3;2] μm	SSA m^2^/kg
Control	2.7 ± 0.7 ^a^	1.9 ± 0.2 ^a^	3270 ± 408 ^c^
Mayo + OLE	2.1 ± 0.0 ^b^	1.7 ± 0.0 ^b^	3663 ± 59 ^b^
Mayo + Alg/Pec	1.8 ± 0.0 ^b^	1.5 ± 0.0 ^c^	4272 ± 9 ^a^

Values are means ± SD. Different superscript letters in the same column indicate significant differences (*p* < 0.05).

**Table 2:** Herschel–Bulkley viscosity parameters of the mayonnaise samples.

	τ_0_ (Pa)	*n*	K (Pa s^n^)
Control	78.8 ^b^	0.3 ^b^	71.3 ^a^
Mayo + OLE	136.1 ^a,b^	0.5 ^a,b^	21 ^b^
Mayo + Alg/Pec	171.4 ^a^	0.7 ^a^	11.4 ^c^

Values are the median ± SD. The flow parameters are the yield stress *τ*_0_ (Pa), consistency index K (Pa s^n^), and flow index *n*. Different superscript letters in the same column indicate significant differences (*p* < 0.05).
